# Information Theoretic Metagenome Assembly Allows the Discovery of Disease Biomarkers in Human Microbiome

**DOI:** 10.3390/e23020187

**Published:** 2021-02-02

**Authors:** O. Ufuk Nalbantoglu

**Affiliations:** 1 Department of Computer Engineering, Erciyes University, 38039 Kayseri, Turkey; nalbantoglu@erciyes.edu.tr; Tel.: +90-352-437-9315; 2 Genome and Stem Cell Center, Erciyes University, 38039 Kayseri, Turkey

**Keywords:** microbiome, metagenomics, genome assembly, biomarker discovery

## Abstract

Quantitative metagenomics is an important field that has delivered successful microbiome biomarkers associated with host phenotypes. The current convention mainly depends on unsupervised assembly of metagenomic contigs with a possibility of leaving interesting genetic material unassembled. Additionally, biomarkers are commonly defined on the differential relative abundance of compositional or functional units. Accumulating evidence supports that microbial genetic variations are as important as the differential abundance content, implying the need for novel methods accounting for the genetic variations in metagenomics studies. We propose an information theoretic metagenome assembly algorithm, discovering genomic fragments with maximal self-information, defined by the empirical distributions of nucleotides across the phenotypes and quantified with the help of statistical tests. Our algorithm infers fragments populating the most informative genetic variants in a single contig, named supervariant fragments. Experiments on simulated metagenomes, as well as on a colorectal cancer and an atherosclerotic cardiovascular disease dataset consistently discovered sequences strongly associated with the disease phenotypes. Moreover, the discriminatory power of these putative biomarkers was mainly attributed to the genetic variations rather than relative abundance. Our results support that a focus on metagenomics methods considering microbiome population genetics might be useful in discovering disease biomarkers with a great potential of translating to molecular diagnostics and biotherapeutics applications.

## 1. Introduction

Microbiome science has transformed the landscape of medicine by introducing novel associations of commensal microorganisms with human health. Hosting trillions of microbes in a broad range of biodiversity, the human body maintains a delicate homeostasis with its microbiota community. Certain shifts in this ecosystem have been associated with health disorders in either causal or consequential manner. The introduction of omics sciences accelerated these discoveries by providing a vast amount of genetic data. One such science field is metagenomics [1], which enables culture-free investigation of environmental samples using next-generation sequencing technologies. Data driven approaches, mastered by bioinformatics algorithms, are adopted to test disease association hypotheses and to discover novel biomarkers. Several disorders, not limited to but including inflammatory bowel disease [2], atherosclerotic cardiovascular disease [3], type 2 diabetes [4], colorectal cancer [5], obesity [6], rheumatoid arthritis [7], liver cirrhosis [8], nonalcoholic fatty liver disease [9], asthma [10], Parkinson’s disease [11], Alzheimer’s disease [12], autism [13], schizophrenia [14] have been associated with gut microbiome in the last decade. Moreover, it was recently shown that tumor and circulating blood microbiomes can distinguish 33 types of cancers as well as different stages of cancer types [15]. Translational metagenomics is expected to promise molecular diagnostics and biotherapeutics solutions to a wide spectrum of chronic diseases [16,17].

Although metagenomics is an active area of research and a great number of bioinformatics tools to explore compositional and functional properties of microbiomes exists, it remains difficult to disentangle the sheer amount of complex metagenomics data. A conventional framework for metagenomics studies involves assembling the genomic content or employing comparative metagenomics over the reference material assembled previously. Metagenome assembly is a challenging problem due to the uneven coverage and mosaic structure caused by the coexistence of close organisms [18]. While great portions of genetic material might fail to assemble into contigs, the generated sequences might also contain a significant fraction of chimeric fragments [19]. Consequently, even though an ample volume of metagenomics data has been sequenced to date, a good amount of these is left as “microbial dark matter” [20,21]. Novel approaches of metagenomic contig generation not following the lead of this convention are potentially important for biomarker discovery tasks.

Quantitative metagenomics, also known as comparative metagenomics, more importantly relies on the abundance structure of compositional and functional elements discovered. That is, binning sequencing reads in taxonomic groups or in genes/metabolic pathways, subsequently using the representation ratio of each unit as a sole feature is the quantification method followed. It is undeniable that this technique has been extremely useful. In fact, almost all microbiome associations reported in the literature are based on relative abundance quantification. However, it is obvious that the relative abundance quantification of a representative unit does not take care of the information contained in different alleles and genetic variants. Accumulating evidence shows that microbial genetic variants might be significantly associated with host phenotypes and they should not be overlooked. Zeevi et al. studied the large structural variants in two geographically distant cohorts. They showed consistent correlations between multiple traits of host health and the existence of the structural variants [22]. Chen et al. discovered characteristic single nucleotide polymorphisms of *Bacteroides coprocola* in type 2 diabetes patients [23]. Genetic variants in microbiota were also associated with various phenotypical aspects of the host such as modulation of immune responses [24], bioconversion of compounds associated with atherosclerosis pathogenesis [25], drug metabolism [26], and dietary habits [27].

Lack of computational methods focusing on variant loci of metagenomes drive researchers to conduct investigations in a projected space of references generated by generic analysis tools. Wang et al. addressed this problem by proposing subtractive assembly methods. In this approach, only the reads containing k-mers varying between host phenotypes are used for metagenome assembly [28,29]. They showed that subtractive assembly scheme results in novel host phenotype associated contigs and genes undiscovered by the conventional metagenome assembly methods. Subtractive assembly provides relevant contigs ignored by conventional assemblers. The adopted assembly algorithm is canonical which prefers consensus sequences over interesting variants. Additionally, they preferred relative abundance-based quantification for disease classification. This inherently suppresses the information that could be exploited from differential genetic variants.

We propose an information theoretic methodology for discovering metagenomic biomarkers associated with a given host phenotype, taking genetic variations carefully into consideration. Our approach scores each building unit (i.e., k-mers) of metagenome assembly by their self-information content associated with the target phenotype. The self-information content is calculated using the probability of each site inferred from statistical tests of phenotype differentiation. These scores supervise the assembly process to infer maximally informed (i.e., maximum average self-information across the phenotype, implying a certain differentiation power) metagenomic fragments. The resulting sequences are expected to artificially contain the available discriminative variants, that we coin as *supervariant fragments*. We tested our methodology on a set of simulated metagenomes and on two different disease-healthy control cohorts of colorectal cancer (CRC) and atherosclerotic cardiovascular disease (ACVD) and showed that it effectively discovers novel disease biomarkers.

## 2. Results

We evaluate the disease classification ability of supervariant metagenomic fragments on a set of simulated and two real-life microbiome studies. Simulated datasets were artificially populated from the genomes of 5 known species with varying compositions. A gut microbiome cohort of CRC patients [30] with 74 patient samples and 53 healthy control samples was considered as the first study case (ENA project number: PRJEB24748). The patients were gathered from all stages of cancer according to using the tumor, node, metastasis staging system. Secondly an ACVD cohort [3] with 218 patients (defined as ≥50% stenosis in one or more vessels) and 187 healthy controls was included in the analyses (ENA project number: PRJEB21528).

### 2.1. Simulated Datasets

In order to observe the genetic variant exploitation capability of the proposed method, we created two-class datasets to be discriminated against. For each dataset, a pair of parameters decided the difference of the classes: *compositional unbalance* and *strain-level unbalance*. Two known strains represented each of the five species in the simulated metagenomes. Compositional unbalance represents the prior distribution odd ratio of the species in both classes (e.g., if the value is 0.5, the species are distributed identically in both classes and if this ratio is closer to 1, each class is likely to be composed of from different species.). Strain-level unbalance is similar to compositional unbalance but it is the odd ratio of strain priors within a species (e.g., if the value is 0.5, both strains of a species are equally likely to be observed in the samples of both classes and if this ratio is closer to 1, completely different strains of a species are likely to be observed in different classes.). *Ferroplasma acidiphilum* strain Y (RefSeq accession no: GCF_002078355.1) and strain DSM 28986 (RefSeq accession no: GCF_013133875.1), *Lactobacillus gasseri* strain ATCC 33323 (RefSeq accession no: GCF_000014425.1) and strain 4M13 (RefSeq accession no: GCF_002158885.1), *Pediococcus pentosaceus* strain ATCC 25745 (RefSeq accession no: GCF_000014505.1) and strain SL001 (RefSeq accession no: GCF_007923185.1), *Prochlorococcus marinus* strain NATL1A (RefSeq accession no: GCF_000015685.1) and strain MIT9313 (RefSeq accession no: GCF_000011485.1), *Streptococcus thermophilus* strain LMD-9 (RefSeq accession no: GCF_000014485.1) and strain ATCC 19258 (RefSeq accession no: GCF_010120595.1) were used for the simulated datasets. MetaSim (version 0.9.1) [31] was used to simulate reads from the genomes and to generate the metagenomes. For each class, 50 metagenome samples with 5 million simulated reads were generated.

**Figure 1:**
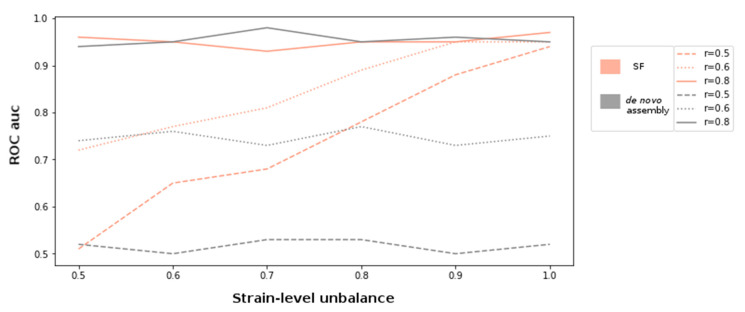
Disease classification performance on simulated datasets with varying compositional unbalance (r). For each r value of 0.5, 0.6, and 0.8, metagenome sets with strain-level unbalance differing between 0.5 and 1 was generated.

For the simulation experiments, metagenomes with compositional unbalance values (r) 0.5, 0.6, and 0.8 were generated. In order to introduce varying genetic diversity at different compositional levels, each setting was also sampled at varying strain-level unbalance parameters ranging between 0.5 and 1. The discovery of supervariant fragments and the classification of the samples were conducted as described in the methods. In parallel, each metagenome was assembled and the ORFs are detected using MEGAHIT assembler and Prodigal gene finder [32]. After evaluating the gene relative abundance values using BWA mapper, sample classification with XGBoost classifier was performed as described in Section 4.6. The performance of each simulated metagenome set in terms of receiver operating characteristics (ROC) area under curve (auc) values is shown in Figure 1. According to this setting, the variation information exploited by supervariant contigs provides an incremental discriminative power as the genetic variance between different metagenome classes are more differential (i.e., with an increasing regime of strain-level). This trend can be clearly observed when the compositional differences are low (r = 0.6) or lacking (r = 0.5). The supervariant fragments did not bring any additional discriminative power when the metagenome classes were already distinct enough regarding composition (r = 0.8). This is an implication that the SFs are especially valuable when the genetic variants, that are not able to be detected by the conventional metagenomic analysis approaches, are the main driver of the microbiome differences. Yet, if phylogenetically distant compositional differences are dominant factor of microbiome differences, SF might not bring any additional power to the analysis.

### 2.2. Comparison of Supervariant Contigs with Metagenome Assembly

After splitting each dataset with 80%–training-20% test partitioning, both training datasets were subject to supervariant metagenomic fragment assembly on scored de Bruijn graphs constructed from filtered k-mers of length 50. The colorectal cancer dataset consisted of 2,347,986,480 distinct k-mers, where the number of total distinct k-mers was 1,958,756,163 for the cardiovascular disease dataset. After quality filtering and removing the low abundance and low prevalence k-mers, the number of k-mers reduced to 355,145,775 for colorectal cancer and to 305,587,338 for cardiovascular disease metagenomes. Since our objective is to discover strong and compact biomarkers of the related phenotypes, we concentrated on the highest scoring supervariant contigs and included the top 10% score range percentile in the analyses.

An investigation of how the current methods are capable of discovering the supervariant regions requires searching for the detected markers in the assembled metagenomes. We used different assembly approaches to observe this in a spectrum. Two of them were popular metagenome assembly tools MEGAHIT [33] and IDBA-UD [34]. Concurrent Subtractive Assembly (CoSA) [29] and Subtractive Assembly (SA) [28] are supervised assembly tools that filter the reads significantly varying between the phenotypes. All assemblies were performed with the same k-mer length of 50. Default values were used for the other parameters of each assembler. We attempted to align the supervariant contigs against the metagenome assemblies using Blastn tool. A minimum alignment coverage of 75% and minimum Blastn similarity score of 90% were considered as the detection criteria. Table 1 shows the ratios of detected supervariant regions out of 336 CRC and 221 ACVD contigs. Conventional metagenome assembly appears to leave more than half of the discovered supervariant regions unassembled or constructed them without interesting variants. Subtractive assembly approaches were observed to assemble most of the supervariant regions. This could be attributed to their supervised approach that reduces the read sets to the subset containing k-mers significantly varying between the phenotypes. However, they are yet not able to discover the entire set of supervariant contigs.

### 2.3. Evaluation of the Biomarkers for Disease Classification

Since the discovered supervariant contigs are fragments of clustered genetic variants or differential abundance regions, they are expected to be associated biomarkers of disease. Validating this hypothesis requires building predictive models on the biomarker candidates and testing the classification performances. A microbiome sample was represented by the k-mer abundance profile along a supervariant fragment. The resulting coverage vector is expected to constitute the features of differential variance, thus classify the samples. The representative vectors in the test set were used to train a machine learning model following 5-fold cross validation on this set. A stochastic gradient boosting classification model (XGBoost, version 0.90 [35]) was used in dropouts meet multiple additive regression trees (DART) booster with binary logistic regressor. Each supervariant contig was tested on the validation set of disease cohorts using a separately trained model.

**Figure 2:**
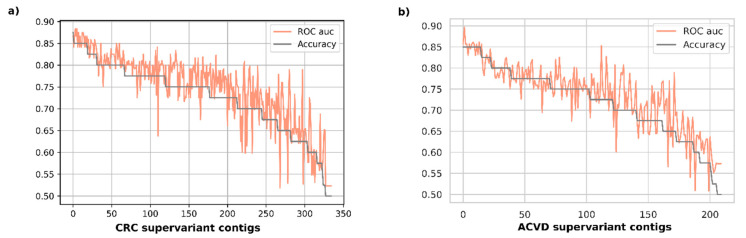
Disease classification performance on the test datasets for CRC and ACVD cohorts are given. Each SF is evaluated independently with a separate model. Disease classification performances in terms of accuracy and ROC auc are presented in descending order of test classification accuracies. (**a**) Classification performance for CRC cohort; (**b**) Classification performance for ACVD cohort.

Figure 2 represents the validation performance of disease classification for each supervariant contig in accuracy-descending order. It was observed that the most accurate 19 biomarkers were all achieving an average accuracy over 0.85 with the top accuracy being 0.875 for CRC validation set. The area under ROC curve scores were attaining all over an average of 0.84 auc, with the top one being 0.883 for the same disease set. The top-15 most accurate biomarkers for ACVD had all average 0.85 accuracy, also attaining an average over 0.833 auc with the maximum one reaching 0.896.

The most discriminative 10 biomarker hits and the associated taxonomic assignments for both datasets are shown in Table 2. While these supervariant fragments perform close to each other, their functional assignments imply essential roles in certain bacterial groups. CRC fragments are observed to be mainly coming from *Clostridia* class, *Lachnospiraceae* and *Erysipelotrichaceae* families. ACVD fragments were found to be of a more diverse origin of taxa, yet with assignments to major functions. Some significant fragments were not e assigned either to taxa or protein functions. These misassignments might be because of being located in intergenic loci or they might be divergent sequences that cannot be detected by eggNOG mapper of BlastX.

### 2.4. Supervariant Contigs vs. Relative Abundance Based Comparative Metagenomics

Conventional quantitative metagenomics studies rely on classifying phenotypes over abundance profiles compiled by mapping the metagenomic sequencing data on comprehensive gene catalogs. Therefore, it is not expected to utilize the discriminative features related to small or medium scale genetic variants (e.g., SNPs, small indels, short tandem repeats etc.). To compare the accuracy of supervariant contigs and abundance-based comparative metagenomics, as well as to observe the discovery characteristics of these two approaches, we extracted the abundance profiles of CRC and ACVD datasets. Around 10 million (pan)genes from the Integrated Gene Catalog of Human Gut Microbiome (iGC) [36] were employed for mapping the sequencing reads. Normalized abundance vectors were used for XGBoost training using the same approach and the same train-test split datasets with supervariant contig biomarkers. The feature vectors constructed from supervariant fragments were concatenated for the top-10 markers based on their information scores, and for the entire marker sets to join and use them as combinatorial markers. Table 3 shows the competitive classification performance of each setting. The combination of supervariant contigs improved the performance of single contigs with a small margin, which might mean the discovered features are correlated.

Relative-abundance and supervariant fragment-based disease classification approaches performed closely for the disease cohorts considered. This does not necessarily mean both methods discover the same markers. The feature importance utility of XGBoost library attributed the majority of the abundance-based model performance to 4 features for CRC dataset, that are genes specific to *Fusobacterium nucleatum*, *Parvimonas micra*, *Solobacterium moorei*, and *Peptostreptococcus* genus. Among the supervariant markers, only one was assigned to *Fusobacterium*. The frequent supervariant contig discoveries from *Clostridia*, *Lachnospiraceae* and *Erysipelotrichaceae* taxa did not have a correspondence in abundance-based feature discovery. In fact, the abundance difference between disease cases and controls were not statistically significant (Mann–Whitney u-test, *p* > 0.05 for all three groups). For the ACVD cohort, following the same methodology, co-discovery of both approaches was observed in taxa *Eubacteriaceae*, *Clostridia*, and *Faecalibacterium*.

**Figure 3:**
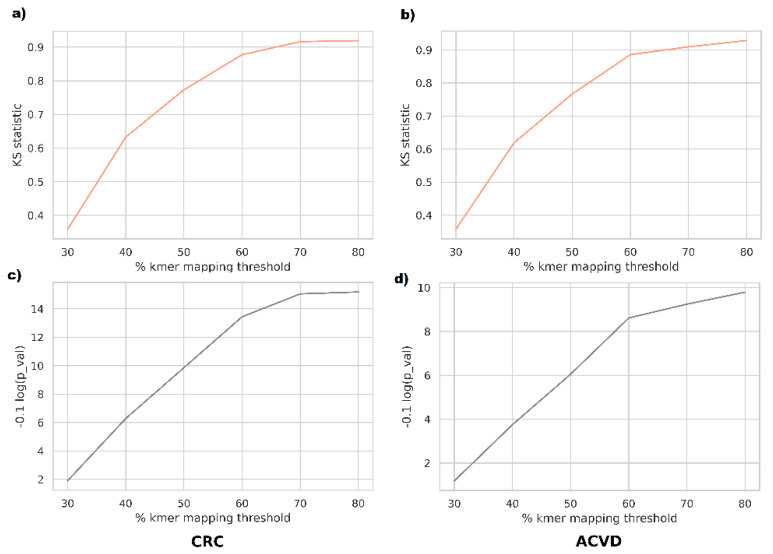
The disease classification accuracy distribution of supervariant fragments and the differential abundance of supervariant fragments are compared using the Kolmogorov–Smirnov Test, with varying mapping thresholds. Supervariant fragment accuracy was found to be significantly greater for all settings. (**a**) Kolmogorov–Smirnov Statistic and (**c**) the test *p*-values in logarimic form for CRC dataset. (**b**) Kolmogorov–Smirnov Statistic and (**d**) the test *p*-values in logarimic form for ACVD dataset.

For a closer investigation of whether the phenotype discrimination ability of supervariant fragments is informed by differential relative abundance or by genetic variants, we performed abundance-based classification tests on the supervariant contigs. In order to map the metagenomic reads to the supervariant sequences, we used a k-mer based mapper. According to this, a read is mapped onto a reference if more than *mapping threshold %* k-mers of the read exist in the reference. Mapping ratio threshold was varied between 30% to 80% to observe the difference in a range of sensitivities. The reason for employing k-mer based mapping was that short-read mappers such as BWA are tuned for highly specific mappings and frequent variants inserted in supervariant regions might result in missing the alignments. Supporting this view, the majority of the taxa corresponding to supervariant contigs were not differentially abundant in the disease cohorts in general. Classification based on those supervariant contigs turned out to perform poorly on average. The populations of classification accuracy scores for relative abundance and genetic variation features were compared using Kolmogorov–Smirnov test. For the range of mapping sensitivities, the variants were observed to be sampled from distributions denser in higher accuracies (Figure 3).

## 3. Discussion

**Figure 4:**
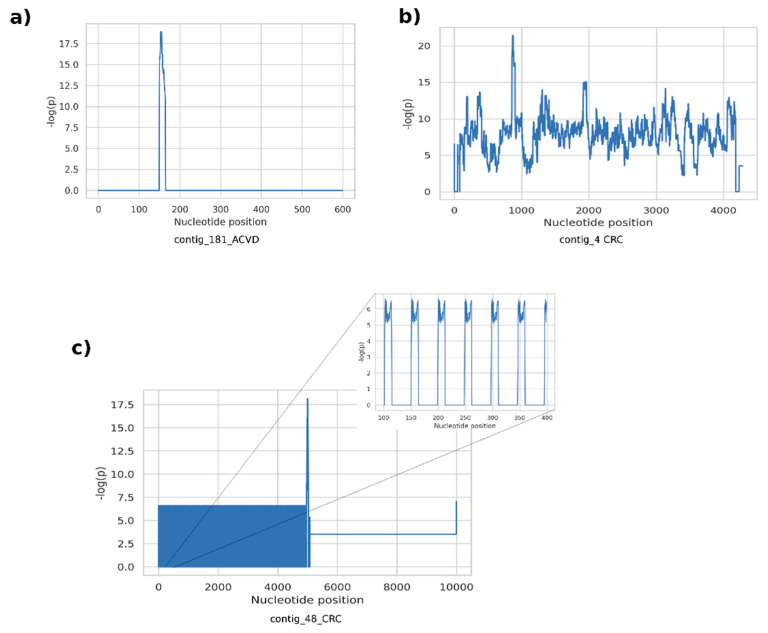
Examples of position-wise self-information scores for the inferred fragments. (**a**) a fragment with a short variance surrounded by regions conserved through the host phenotypes. (**b**) A discriminative island representing either a differential abundance or a large structural variant region. (**c**) a fragment with periodic hypervariant sequences, reflecting as spike trains to the position-wise self-information scores.

The proposed methodology for the discovery of metagenomic regions exhibiting differential variation between different host phenotypes is motivated by the rationale that current abundance-based coarse discovery paradigm in quantitative metagenomics is inherently unable to resolve detailed information. Not only the quantity/existence of a taxon or a functional element is associated with the host phenotype, but the genomic landscape and the genetic variations are also likely to contribute to the associated mechanisms. Here, we attempt to emphasize such structures by assembling potentially artificial metagenomic fragments with an assembly algorithm guided by the phenotype information. Our observation on the fragments generated from the examined datasets revealed that supervariant contigs emphasize three main different types of differential events. A first class of fragments included short genetic variants such as SNPs and short indels, resulting in short contig assemblies. Typically, the position-wise self-information scores consist of narrow spikes surrounded by non-informative (i.e., conserved throughout host phenotypes) regions (Figure 4a). Low, or almost zero self-information content around the self-information peaks implies that the average abundance of these contigs is not differentially significant, whereas the short variants are significantly different for different phenotypes. These short variants are discriminative features contributing to the classification using the proposed classification scheme. On the other hand, these variants are ignored, and the self-information content cannot be exploited by the conventional comparative metagenomics methods. The second type of fragments typically contain high-information islands throughout a contig, with plateaus of high scores on the position-wise self-information score graphs (Figure 4b). These islands can either stem from differential abundance of genomic content or they might harbor large structural variants differing between host phenotypes. Unfortunately, it is hard to distinguish these two phenomena unless non-chimeric long contigs or reference genomes are available. A third common class of supervariant fragments consists of short periodic variances associated with host phenotypes, that translate to periodic spike trains in position-wise self-information score graphs (Figure 4c). Such cases indicate that tandem repeats or short oligonucleotide repeat regions are genomic features associated with related host phenotype functions.

We reported that for the majority of the supervariant fragments, the differential abundances between the disease phenotypes are not discriminative. Therefore, their disease associations can be mainly attributed to the genetic variations. In fact, a good portion of the inferred contigs are not generated by conventional metagenome assembly. Even if that was achieved, supervariant fragments would not be emphasized as biomarkers due to lack of significant differential abundance. Current convention on comparative metagenomics, on the contrary, considerably depends on abundance of taxonomic and functional units [37]. We believe that it might be fruitful to integrate genetic variation factors in comparative metagenomics studies. Novel and stronger microbiome–host phenotype associations are likely to be achievable with such a perspective. It is straightforward to incorporate the relative abundance vectors and the supervariant contig representations as additional features to disease classification algorithms. Downstream analysis such as feature selection might be useful to assign importance to genetic variance and abundance difference combinations.

Our analysis on real-life data revealed that the proposed method promises a better improvement for the CRC dataset while the improvement for the ACDV data seems insignificant. The underlying reason for this might be differing genotypical drivers for the disease phenotypes. CRC microbiome might have significant genetic variants associated with the disease, while the variants in ACVD context might be insignificant or correlated with the composition. Further rigorous studies on characterizing such phenomena can be especially important to illuminate the microbiome structure in different disease phenotypes.

Annotation studies on the discovered fragments left some fragments uncharacterized without a functional or a phylogenetic assignment with both homology search methods employed. An uncharacterized sequence might be of intergenic regions, since eggNOG Mapper, BlastX, and Kaiju all use protein sequences as the search databases and noncoding regions are not available to be hit. Another possibility is the unavailability of the discovered markers in the molecular databases, as a great amount of metagenomic dark matter remains to be undiscovered [38]. In addition, it should be noted that the assembled sequences are artificial contigs with enriched variations, which might not be harbored all in a certain strain. Thus, resulting supervariations might not be detectable with the close-homology search tools mentioned.

The proposed algorithm populates the most relevant metagenomic sequences of specific loci in a one-dimensional data structure (i.e., a sequence). This approach is biologically relevant, and physical entities of biomarkers can be synthesized into DNA fragments to be utilized as biotechnological products such as diagnostic/therapeutic kits. However, the nature of fragment assembly forces the inferred product to pick a winner (i.e., most relevant variant) for each locus, eliminating the other minor variants carrying less self-information. For this reason, it might be interesting to propose more informative and complex data structures, such as profile hidden Markov models, to represent the metagenomic entities associated with host phenotypes. We believe the supervariant metagenomic loci concept is open to investigation of useful bioinformatics tools.

The supervariant contig assembly algorithm is based on a naïve Viterbi decoder, without any constraints for chimera control, or read guided traversal or scaffolding of de Bruijn graphs. For this reason, the generated contigs might be vulnerable to chimera. In case of transforming the biomarkers to the subjects of targeted sequencing agents, validation of the fragments is needed. PCR-based positivity tests should be downstream steps of biomarker discovery using the methodology proposed in this work. Nevertheless, in silico investigations might still be utilizable even though the contigs were chimeric, as they solely serve as computational models.

According to our results, combinatorial use of supervariant fragments only brings a marginal improvement to disease classification performance. Moreover, the discovered contigs appear to cluster in certain taxonomic groups rather than ranging in a diverse phylogenetic tree. Unfortunately, it was not possible to assign the fragments to lower taxonomic ranks to decide if they are of the same origin, because they were mainly observed in multiple species. Taking these observations into consideration, it is likely that these variations are coexisting/correlating. Whether specific stains of certain microorganisms are associated or not is a natural question to be raised at this point. Pioneering studies report genetic events at microbiome level such as recombination, oligocolonization, and adaptations are associated with host genotypes [39]. Metagenomic studies regarding the population genetics of microbiome communities might be considered as further investigation of the variants in metagenomes in human disease context.

Another branch of further investigations motivated by the results of this work might be the study on functional consequences of the variants associated with the host phenotypes. Observational studies on the regulatory effects on gene expression or taxa abundance or structural studies investigating the changes in protein structure/function on the related variants might be conducted. Similarly, interventional experiments introducing variant microbiomes in vivo are potentially valuable.

The discovered biomarkers could be employed in developing next-generation sequencing based laboratory procedures for microbiome-based diagnostics of related diseases. A further investigation of variant sequences distinguishing the patients from the healthy controls could help disease etiology or prognosis studies via designing interventional in vivo experiments.

## 4. Materials and Methods

We introduce an ab initio approach, diverging from the top-down perspective of quantitative metagenomics, where pipelines of metagenome assembly, functional and taxonomic assignment, and biostatistics/ML-based host phenotype association are adopted. We start by determining the associations on raw data and follow the assembly step guided with this information in order to direct attention on the biomarker candidates by construction. Below, the underlying idea and the steps of this procedure are provided.

### 4.1. Supervariant Contigs: Genomic Sequences Containing Maximum Average Self-Information

In order to define an information theoretic framework for inferring DNA sequences descriptive of a related phenotype, we introduce a few practical definitions here. To assess quantitative measures, firstly we need standard units of metagenomics data. We choose quantities of short oligonucleotides of fixed-length k (i.e., k-mers) defined as:

(1) $$O_{i}\in K=\left\{AAA..AA,AAA..AC,\ldots TTT..TG,TTT..TT\right\},\|K\|=4^{k}.$$

Assume that a k-mer harbors a genotypical motif associated with a phenotype. In case, given two different phenotypes $F_{1}$ and $F_{2}$ e.g., disease vs healthy) is expected to result in differing distributions $f\left(g_{i}|F_{1}\right)$ and $f\left(g_{i}|F_{2}\right)$. Here, $g_{i}$ represents the relative frequency of k-mer $O_{i}$ in a metagenome. As this phenotype association gets stronger, the uncertainty of *O_i_* ’s relative frequency given a phenotype would be lower. That is to say, a self-information metric can quantify the genotype-phenotype association strength. Let us define the self-information of a k-mer *o_i_* as $I\left(O_{i}\right)$ which measures how certain it is to describe the phenotype from the relative frequency of $O_{i}$.

As an approximation of this self-information measure, we can utilize the statistical difference in the probability distributions of $f\left(g_{i}|F_{1}\right)$ and $f\left(g_{i}|F_{2}\right)$ determined from a hypothesis test:

(2) $$I\left(O_{i}\right)\propto -\log\left(p_{O_{i}}\left(H_{0}\right)\right).$$

where given a statistical hypothesis test and empirical data, $p_{O_{i}}\left(H_{0}\right)$ is the probability that *o_i_* kmer follows similar distribution parameters across phenotypes. As the distribution between the phenotype conditions differ, the defined metric will have a larger value, in accordance with self-information. The probability, $p_{O_{i}}\left(H_{0}\right)$ is calculated by a statistical test on the empirical data where the relative frequency of *O_i_* for a phenotype *F* is considered as a sampling instance of $f\left(g_{i}|F\right)$. A hypothesis test for the similarity of $f\left(g_{i}|F_{1}\right)$ and $f\left(g_{i}|F_{2}\right)$ distributions are evaluated over the corresponding k-mer relative frequencies, and $p_{O_{i}}\left(H_{0}\right)$ is determined by the *p*-value of this testing. We adopted Mann–Whitney U test for our experiments, since a non-parametric test would be more appropriate for the uncharacterized distributions of metagenomics data.

According to metagenome assembly problem, an achievable path in the de Bruijn graph constructed from the k-mers of the sequencing data is considered as a metagenomic contig candidate,

(3) $$t_{j}\in \left\{\forall achievablepathsofG\right\},t_{j}=o_{1_{i}}o_{2_{i}}\ldots o_{m_{i}}.$$

annotated with *m* k-mers $t_{ĵ}$ traverses. Among all achievable paths, we define the ones containing maximum average self-information $t_{masi}=t_{ĵ}$ such that:

(4) $$\hat{j}=\underset{j}{argmax}\frac{1}{\left|t_{j}\right|}\sum_{l=1}^{\left|t_{j}\right|}I\left(O_{l_{j}}\right)=\underset{j}{argmin}\frac{1}{\left|t_{j}\right|}\sum_{l=1}^{\left|t_{j}\right|}\log\left(p_{O_{l_{j}}}\left(H_{0}\right)\right).$$

Clearly, $t_{masi}$ will contain maximal number of interesting genetic variants carrying information about the corresponding phenotype, thus forming a supervariant contig. Equation (4) evaluates a genome fragment based on its average self-information content. Here, the motivation is that contigs harboring discriminatory sections of variants associated with the phenotype should be assembled. It is possible to define other self-information metrics concentrating on single/local variants other than the average of a contig. Our experiments showed that the average self-information scoring is more successful in detecting discriminatory supervariant contigs (Supplementary Materials). Therefore, we adopted this scheme. We address this optimization problem and introduce a feasible solution of inferring supervariant contigs next.

### 4.2. Selecting a Discriminative Subset of k-Mers

In order to focus on differential metagenomic fragments that emphasize a targeted phenotypical difference within a cohort, the first step is determining the significant k-mers within the raw sequencing reads. k-mers are the spectrum of all short oligonucleotides of a fixed size, observed in a set of genomic sequences. The record of fixed size oligonucleotides is preferred in a range of sequence analysis tasks for practical purposes, as efficient data structures allow their lightweight processing. Given the preferred oligonucleotide length (i.e., the parameter k) is long enough to specifically point a certain genomic location, each k-mer harbors useful information reflecting the characteristics of the corresponding region. The insertion-deletion of large genomic regions are detectable by the existence of k-mers mapping onto that region. Other variants, such as single nucleotide polymorphisms and short insertions/deletions also result in alternating k-mers. Moreover, when the quantity/copy number of a genomic region is indicative of a condition, k-mer count records inherit the abundance profile of the corresponding regions. Therefore, representing genomics data with specific k-mer count profiles has significant power to capture characteristics in both genetic variance and differential abundance of metagenomic samples.

We represented the metagenomic samples with k-mers of length 50. Due to the sheer size of metagenomic datasets, a highly efficient k-mer count program, KMC 3 [40] was employed. As the default bit allocation for a single k-mer count (255) saturated quickly, we set the count flag cs as 65,535. Due to instrumental imperfections in next-generation sequencing, several base miscalls are observed in a sequencing dataset. This noise, resulting in tremendous amount of singleton k-mers were removed by quality filtering option of KMC 3 by trimming/filtering sequencing regions under Phred quality score of 30. Downstream filtering of k-mers in abundance and prevalence also applied. Any k-mer observed less than 10 times in a metagenome sample were removed, in order to filter out low-abundance/rare genetic material, as well as focusing on genomic regions with lower probability of sequencing errors. k-mers observed in less than 10% of the entire samples were also removed as low prevalence material, to avoid nongeneralizable biomarkers. The regarding stages of filters also serve for practical purposes minimizing the memory and processing burden significantly to the feasible ranges.

Differential k-mers, varying significantly in relative quantity between disease and healthy control phenotypes were quantified in a univariate fashion performing Mann–Whitney U-test on each k-mer independently. Each k-mer obtained a self-information of phenotype association score $i_{k}=-\log p_{k}$ where $p_{k}$ is the *p*-value of k-mer k calculated from the statistical tests.

An 80%–20% train-test set split was used to avoid information leakage, and the k-mers were scored on the training set.

### 4.3. Constructing the Supervariant Metagenomic Fragments

The self-information of phenotype association scores for each k-mer implies a differential abundance or genetic variant between the disease and healthy phenotypes in the locus of that k-mer. Although the scores are calculated by univariate statistics assuming independence, a discriminative metagenomic fragment should contain clusters of k-mers with high scores. Our approach to discover these informative regions is employing fragment assembly informed by the scores.

Current approach to (meta)genome assembly is based on constructing de-Bruijn graphs from the k-mers observed in the sequencing data, and traversing Eulerian paths on these graphs [41,42]. However, metagenomic assembly is challenging due to uneven coverage issues. It has been reported that sequences that might harbor importance as biomarkers might remain unassembled in canonical approach [43]. Furthermore, genome assemblers tend to prefer high coverage content to construct a consensus sequence. In case, non-dominant variants that are associated with interesting phenotypes are overlooked and the related information is lost.

**Figure 5:**
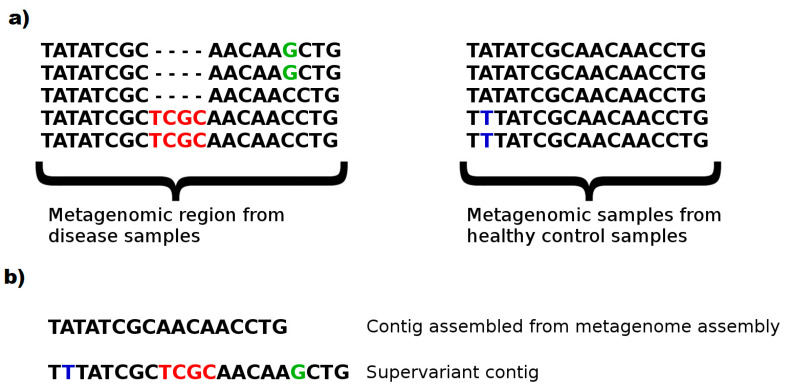
A toy example showing the genomic loci with genetic variances associated with host phenotypes (**a**). The resulting metagenomic contig obtained by conventional metagenomic assembly and a supervariant contig which populates the genetic variants that are informative with respect to the targeted phenotypes (**b**).

This phenomenon can be clearly seen in Figure 5. A subset harboring an insertion region (2/5 samples with TCGC insert) and a substitution (2/5 samples with C > G substitution) of disease phenotypes, as well as a substitution (2/5 samples with A > T substitution) of healthy controls are typical to the phenotype. This knowledge would enable evaluating a sample and classifying the correct phenotype with a relatively accurate decision. However, a (meta)genome assembler would prefer the most frequent k-mers and construct a consensus fragment lacking the biomarker variants. Note that, even a metagenome assembler considering multiple strains is likely to ignore such variants to prevent chimeric assemblies. On the contrary, without avoiding chimera, and attempting to populate maximal number of discriminative genomic features in a chimeric fragment might be a fruitful approach to construct artificial metagenomic markers with a greater ability to capture phenotypes from microbiome samples. We call such artificial contigs as *supervariant contigs*. Our fragment assembly algorithm attempts to discover the most informative supervariant contigs by inferring the fragments with maximal average self-information of phenotype association.

**Figure 6:**
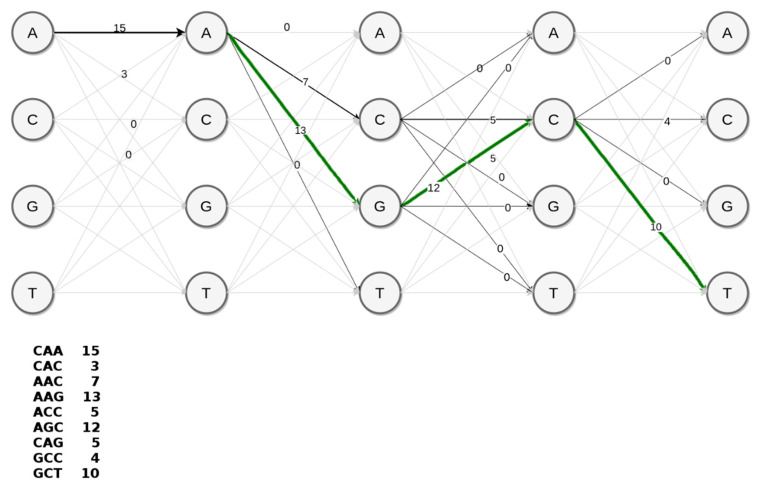
Contig assembly problem from the toy example of Figure 4, represented with a trellis structure. The trimer scores shown below the trellis represent the self-information scores *i_k_* for each k-mer. When these scores, obtained by statistical tests, guide the traversal using Viterbi decoding, the corresponding maximal scoring path corresponds to populating the significant variants in a supervariant fragment, shown as the green path.

Since discriminative k-mers with high scores correspond to marker variants of a supervariant contig, the problem of discovering a supervariant contigs is equivalent to inferring a path within the de Bruijn graph with maximal average score of k-mers traversed. An optimal and feasible solution to this graph problem is dynamic programming. For the ease of representation, we can consider the achievable paths (i.e., possible assemblies) as a trellis such in the simplified example in Figure 6. This toy example represents the construction of AAGCT section of the supervariant contig shown in Figure 5. A conventional assembler prefers the achievable path with the highest coverage edges in a greedy fashion. This would correspond to AACCG preferring abundant k-mer path through C over phenotypically discriminating path through G. However, embedding the corresponding k-mer scores in the edges of the trellis, it is possible to traverse the highest scoring (i.e., most discriminative) supervariant path. Basically, employing Viterbi decoding algorithm [44] provides a simple solution for that. However, unlike the toy example, the choice of k-mer size (could be considered as a 49th order Markov Model) would result in a large trellis. Note that, trellis representation is to show the equivalence of the problem. In fact, the de Bruijn graph with score-weighted edges serves the same purpose with a feasible data-structure in practice. Each node in the de Bruijn graph structure points to the node corresponding to the optimal subsolution. This enables backtracking through the optimal Viterbi path and decoding the supervariant contig accordingly.

The supervariant fragment assembly algorithm generates contigs with fixed sizes of 10 Kbp starting from the maximum scoring seeds, without replacement. The average scores of the supervariant fragments are used for picking the top candidates as biomarkers.

### 4.4. Annotating the Supervariant Metagenomic Fragments

Each supervariant fragment was searched for its origin of taxa and functional properties. A consensus of BlastX search over the entire nt database of NCBI and eggNOG mapper [45] was used to annotate the genes the supervariant fragments are placed in. Both programs are used with the default parameters. Extracted open reading frames were classified in taxa using Kaiju metagenomic classifier [46]. The most plausible assignments were determined by the lowest common ancestor of the BlastX and Kaiju assignments.

### 4.5. Classifying Metagenomic Samples Using the Supervariant Fragments

Constructed set of supervariant contigs are expected to contain discriminative genomic variants or differential abundance regions to be tested with mapping actual test metagenomes for validity. Each sample is characterized for its supervariant contig features in a vectorized representation. For each k-mer of a supervariant contig, the corresponding relative abundance in a sample constitutes the entries of a vector in an orderly manner. For multiple contigs, the vectors are simply concatenated. These vectors are fed to classifier models as inputs. XGBoost classifiers are trained and tested on the same held-out dataset used for k-mer scoring previously. 5-fold cross validation was applied for the classification process.

### 4.6. Quantitative Metagenomics Analysis Using De Novo and Previously Assembled References

For comparison, investigated metagenomes were profiled using two commonly used conventional metagenome analysis approaches [37]. Firstly, each metagenome was mapped onto the integrated reference gene catalog (iGC) of human gut microbiome [36] using BWA short read mapper [45]. Second approach involved de novo assembly and annotation of the metagenome sets. Each metagenome set was assembled using MEGAHIT assembler and the ORFs were detected using Prodigal gene finder [32]. Resulting gene catalogs were used as the reference similar to iGC. Normalized abundances of each gene for iGC and the vector representations explained in Section 4.5 for the de novo assembly catalogue were used as features and the same XGBoost classifier setup with supervariant fragment representations is adopted for training and testing.

## Figures and Tables

**Table 1:** The supervariant fragments (SF) discovered by the proposed method are searched within the contigs assembled by CoSA, SA, MEGAHIT, and IDBA-UD assemblers. The ratio of SFs discovered by each assembly for colorectal cancer (CRC) and atherosclerotic cardiovascular disease (ACVD) are provided.

	SF	CoSA	SA	MEGAHIT	IDBA-UD
CRC	336	87.5% (294/336)	63.3% (213/336)	48.2% (162/336)	34.8% (117/336)
ACVD	211	80.5% (170/211)	72.5% (153/211)	34.6% (73/211)	38.4% (81/211)

**Table 2:** The top-10 supervariant fragments according to their disease classification performances were selected and annotated. The functional and taxonomic assignments are provided.

**CRC**			
**Contig #**	**Function**	**Taxonomy**	**ROC auc**
contig_328	L-rhamnose mutarotase	*Bacteroidia bacterium*	0.867 ± 0.004
contig_227	AAA family ATPase	*Lachnospiraceae*	0.841 ± 0.005
contig_297	hypothetical protein	*Fusobacterium*	0.841 ± 0.006
contig_222	spore germination protein	*Lachnospiraceae*	0.883 ± 0.005
contig_189	-	-	0.867 ± 0.005
contig_164	nucleoside phosphorylase	*Lachnospiraceae*	0.867 ± 0.017
contig_68	phosphoglycerate dehydrogenase	*Coprobacillus* spp.	0.883 ± 0.013
contig_108	nucleoside phosphorylase	*Lachnospiraceae*	0.841 ± 0.013
contig_9	-	-	0.875 ± 0.01
contig_337	DNA-binding protein WhiA	*Clostridiales*	0.841 ± 0.008
**ACVD**			
**Contig #**	**Function**	**Taxonomy**	**ROC auc**
contig_83	COG NOG13196 non supervised orthologous group	*Dorea*	0.861 ± 0.007
contig_111	Leucine rich repeats (6 copies)	*Eubacteriaceae*	0.896 ± 0.006
contig_38	Cysteine-rich secretory protein family	*Clostridia*	0.861 ± 0.003
contig_51	D-alanyl-D-alanine carboxypeptidase	*Blautia* spp.	0.833 ± 0.003
contig_16	-	-	0.861 ± 0.007
contig_57	Ig-like domain-containing protein	*Eubacterium ventriosum*	0.844 ± 0.002
contig_4	-	-	0.861 ± 0.068
contig_12	hypothetical protein	*Faecalibacterium*	0.867 ± 0.004
contig_130	-	-	0.861 ± 0.002
contig_124	hypothetical protein	*Eubacterium ventriosum*	0.867 ± 0.006

**Table 3:** The partial and full set of supervariant contigs are used as combinatorial biomarkers and the overall disease classification performances were compared with differential relative abundance features detected over iGC database and de novo assemblies for CRC and ACVD datasets.

		SF (Full)	SF (Top-10)	iGC	De Novo Assembly
CRC	Accuracy ROC auc	0.895 ± 0.014 0.911 ± 0.009	0.811 ± 0.03 0.82 ± 0.027	0.865 ± 0.009 0.841 ± 0.004	0.857 ± 0.012 0.862 ± 0.008
ACVD	Accuracy ROC auc	0.875 ± 0.008 0.9 ± 0.006	0.79 ± 0.04 0.795 ± 0.024	0.89 ± 0.012 0.92 ± 0.01	0.872 ± 0.006 0.89 ± 0.004

## Data Availability

The microbiome biomarker sequences discovered in this work are available online from https://github.com/nalbant/SupervariantMetagenomicFragments. The Python scripts and the intermediate outputs are available upon request from the author.

## Supplementary Materials

The following are available online at https://www.mdpi.com/1099-4300/23/2/187/s1.

## Abbreviations

The following abbreviations are used in this manuscript:

CRC	Colorectal Cancer
ACVD	Atherosclerotic Cardiovascular Disease
SA	Subtractive Assembly
CoSA	Concurrent Subtractive Assembly
DART	Dropouts meet Multiple Additive Regression Trees
ROC	Receiver Operating Characteristics
auc	Area Under Curve
iGC	Integrated Gene Catalog of Human Gut Microbiome
SF	Supervariant Fragments
SNPs	Single Nucleotide Polymorphisms
PCR	Polymerase Chain Reaction
ML	Machine Learning
ORFs	Open Reading Frames
