# Supplementary material for: Information Theoretic Metagenome Assembly Allows the Discovery of Disease Biomarkers in Human Microbiome
Source: Entropy (Basel). 2021 Feb 2;23(2):187. doi: 10.3390/e23020187 (PMC7913240; doi:10.3390/e23020187)
Supplement: Supplementary file 1 [file entropy-23-00187-s001.pdf]

Article

Version January 11, 2021 submitted to Entropy

The discriminatory variants associated with disease phenotypes might be local features existing in small loci, such as SNPs in the extreme case or they might be clusters of variants observed in genomic fragments pervasively (e.g. large structural variants, islands of mutations, sections of differential relative abundance, etc.). Throughout the supervariant fragment assembly phase, the self-information scoring scheme can intrinsically prefer and emphasize these two different categories, according to the scoring definition.

We have defined two such scoring metrics:

i) Average Self-information (L_2): This metric measures the information content of a genome fragment as the average self-information of the k-mers contained in the fragment. For an achievable path t_j in the de Bruijn graph, the information content of the path is defined as

$$I(t_j) = \frac{1}{|t_j|} \sum_{l=1}^{|t_j|} i(o_{l_j}) = \frac{1}{|t_j|} \sum_{l=1}^{|t_j|} \log(p_{o_{l_j}}(H_0)). \tag{1}$$

The Average Self-information (L_2) is expected to emphasize clusters of variants with an accumulating score.

ii) Maximum Self-İnformation (L_∞): This measure defines the information of a fragments with the most discriminatory k-mer harboured in it as follows:

$$I(t_j) = \arg\max_{l_j} i(o_{l_j}) = \arg\max_{l_j} \log(p_{o_{l_j}}(H_0)). \tag{2}$$

It is expected that L_∞ measure picks the genomic fragments with significant k-mers, emphasizing local features.

Table 1 provides the top discriminatory supervariant fragments assembled by the maximum-self information scheme. We have compared the disease discrimination performance of these two metrics, and observed that scoring based on average self-information significantly outperforms the (L_∞) scoring (Table 2). According to our experiments, strongly discriminatory local variants were not as powerful as regions of variable fragments associated with the diseases. Therefore, we adopted the former scoring scheme in our method.

Table 1. Top-10 supervariant fragments assembled using L_∞ scoring were selected according to their disease classification performances were selected and annotated. The functional and taxonomic assignments are provided.

Contig #	Function	Taxonomy	ROC auc
	**CRC**		
contig_108	nucleoside phosphorylase	*Lachnospiraceae*	0.783∓0.012
contig_79	PLP-dependent aminotransferase family protein	*Lachnospiraceae*	0.731∓0.009
contig_289	- (KEGG: K06921)	*Ruminococcaceae*	0.742∓0.007
contig_238	-	-	0.79∓0.014
contig_41	- (detected EC number: 2.7.7.27, KEGG module: M00565)	*unclassified Lachnospiraceae*	0.734∓0.009
contig_212	family 16 glycosylhydrolase	*Clostridiales*	0.711∓0.004
contig_306	response regulator transcription factor	*Erysipelotrichaceae*	0.77∓0.008
contig_292	- (detected EC number: 2.4.1.21,2.7.7.27)	-	0.706∓0.015
contig_23	HAD hydrolase-like protein	*Lachnospiraceae*	0.722∓0.029
contig_335	hypothetical protein	*Faecalibacterium*	0.69∓0.01
	**ACVD**		
contig_120	-	*Clostridia*	0.817∓0.009
contig_182	holo-ACP synthase	*Clostridiales*	0.822∓0.011
contig_78	-	-	0.793∓0.004
contig_116	phosphoenolpyruvate–protein phosphotransferase	*Blautia spp.*	0.805∓0.006
contig_14	-	-	0.8∓0.018
contig_128	16S rRNA (uracil(1498)-N(3))-methyltransferase	*Blautia*	0.791∓0.008
contig_162	hypothetical protein	*Eubacterium*	0.817∓0.07
contig_54	glycoside hydrolase family 32 protein	*Coprobacillus*	0.802∓0.006
contig_31	-	-	0.78∓0.011
contig_67	cellulase family glycosylhydrolase	*Eubacterium ventriosum*	0.776∓0.002

Table 2. Partial and full set of supervariant contigs are used as combinatorial biomarkers and the overall disease classification performances were compared with differential relative abundance features detected over iGC database for CRC and ACVD datasets.

		**Average Self-information (L_2)**		**Maximum Self-İnformation (L_∞)**	
		**SF (Full)**	**SF (Top-10)**	**SF (Full)**	**SF (Top-10)**
**CRC**	**Accuracy**	0.895∓0.014	0.811∓0.03	0.765∓0.009	0.684∓0.013
	**ROC auc**	0.911∓0.009	0.82∓0.027	0.792∓0.009	0.701∓0.017
**ACVD**	**Accuracy**	0.875∓0.008	0.79∓0.004	0.81∓0.01	0.684∓0.1
	**ROC auc**	0.9∓0.006	0.795∓0.024	0.827∓0.008	0.724∓0.09

© 2021 by the authors. Submitted to *Entropy* for possible open access publication under the terms and conditions of the Creative Commons Attribution (CC BY) license (http://creativecommons.org/licenses/by/4.0/).
